# Pre-operative activity level and a sport or recreation injury mechanism are associated with 2-year clinical outcome after proximal hamstring tendon repair

**DOI:** 10.1007/s00402-026-06252-0

**Published:** 2026-03-12

**Authors:** Jay Ebert, Peter Edwards, Sven Klinken, Brendan Ricciardo, Peter Annear, Peter D’Alessandro

**Affiliations:** 1 School of Human Sciences (Exercise and Sport Science), University of Western Australia, Perth, Australia; 2 Curtin University, Perth, Australia; 3 Perth Radiological Clinic, Perth, Australia; 4 Coastal Orthopaedics, Perth, Australia; 5 Perth Orthopaedic and Sports Medicine Centre, Perth, Australia; 6 Orthopaedic Research Foundation of Western Australia, Perth, Australia

**Keywords:** Proximal hamstring avulsion, Surgical repair, Clinical outcome, Satisfaction, Predictive variables

## Abstract

**Introduction:**

Surgical repair of proximal hamstring tendon ruptures has demonstrated encouraging outcomes and satisfaction rates, superior to non-operative management. This study sought to investigate injury, surgical and post-operative factors associated with clinical outcome and satisfaction 2-years after proximal hamstring tendon repair of acute tendon injuries.

**Methods:**

This study included 59 patients undergoing proximal hamstring repair for acute tendon ruptures. Clinical assessment pre-operatively and 2-years post-operatively included the Perth Hamstring Assessment Tool (PHAT), Lower Extremity Functional Scale (LEFS) and Tegner Activity Scale (TAS). Regression analysis assessed the contribution of pre-operative patient (age, sex, body mass index, TAS), injury/surgery (time from injury to surgery, injury mechanism, semimembranosus and conjoint tendon retraction) and post-operative (peak isokinetic knee flexor strength) variables, to the 2-year PHAT and reporting complete satisfaction.

**Results:**

From baseline to 2-years, the PHAT improved by 47.1 points (95% CI, 41.9 to 52.3; *p* < 0.001), with 97.1% of patients meeting the PHAT minimal important change of 8.6 points by 2 years post-surgery. Furthermore, the LEFS improved by 44.5 points (95% CI, 39.9 to 49.1; *p* < 0.001) and TAS by 2.6 points (95% CI, 2.1 to 3.2; *p* < 0.001). Forty-six (78.0%) patients were ‘very satisfied’ with their 2-year outcome. Univariable analysis indicated that age (*p* = 0.004), baseline TAS (*p* = 0.006), a sport/recreation injury mechanism (*p* < 0.001) and 6-month normalized knee flexor torque (*p* = 0.030) were associated with the 2-year PHAT. In the final multivariable model, only the baseline TAS (*p* = 0.021) and a sport/recreation injury mechanism (*p* = 0.001) remained. No variable was significantly associated with being ‘very satisfied’ with 2-year surgical outcome.

**Conclusions:**

Clinical scores improved significantly from baseline to 2 years after proximal hamstring repair. While pre-operative TAS and a sport/recreation injury mechanism were associated with the 2-year PHAT, no variables were associated with being ‘very satisfied’ with the 2-year outcome.

**Level of evidence:**

Prospective cohort study, Level of evidence 3.

## Introduction

Hamstring injuries are common [1], with about 12% of these injuries resulting in a rupture of the proximal tendon attachment [16]. Non-operative management may be explored [1], particularly for certain pathologies such as single-tendon injuries, 2-tendon injuries with <2 cm of retraction and partial tears [26], though patients may still proceed toward surgical repair due to persistent pain, reduced strength, and limited sports performance [13]. Nonetheless, surgical repair has demonstrated superiority over non-operative management [5], with outcomes more favorable in those undergoing repair for acute (versus chronic) ruptures [5, 9, 13, 14].

Improvement in clinical outcomes, high levels of satisfaction and sports participation, and a low re-rupture rate, has been reported following surgical repair [5, 13, 14]. However, information is lacking on what factors are associated with a good clinical outcome and improved satisfaction after proximal hamstring repair. Recently, it was reported that the post-operative clinical improvement was better in those < 50 years of age versus those > 50 years [20]. However, Best et al. [3] reported that a higher patient-reported functional outcome was associated with being female and a delayed surgery timeframe, though age and the degree of stump retraction on imaging was not. A retrospective study by Bowman et al. [6] could not identify any relationships between patient demographics, medical comorbidities, tear characteristics and repair technique, with clinical outcomes at a minimum 1-year after proximal hamstring repair. With respect to return to sport, a recent review reported that elite athletes are more likely to return when compared with non-elite athletes [7]. A recent study on elite athletes showed that being male, those undergoing surgery for isolated semimembranosus tears, or surgery for complete tendon ruptures without evidence of an empty footprint, were all predictive of a better post-operative level of sporting competition [18].

A robust prospective study investigating the association between patient-related, injury and surgical, and post-operative factors, and patient outcome and satisfaction after proximal hamstring repair is lacking. This information would provide a means of more accurate pre-operative patient education and setting of realistic expectations after surgery. This study sought to investigate which patient and surgical characteristics, as well as post-operative variables, were associated with 2-year clinical outcome and satisfaction after proximal hamstring repair. Firstly, it was hypothesized that clinical outcomes for the cohort would significantly improve from baseline (pre-surgery) to 2 years post-surgery. Secondly, it was hypothesized that certain patient demographics, injury and surgical characteristics, and post-operative variables, would be associated with 2-year clinical outcome and being ‘very satisfied’ with their overall outcome.

## Materials and methods

### Patients

**Fig. 1:**
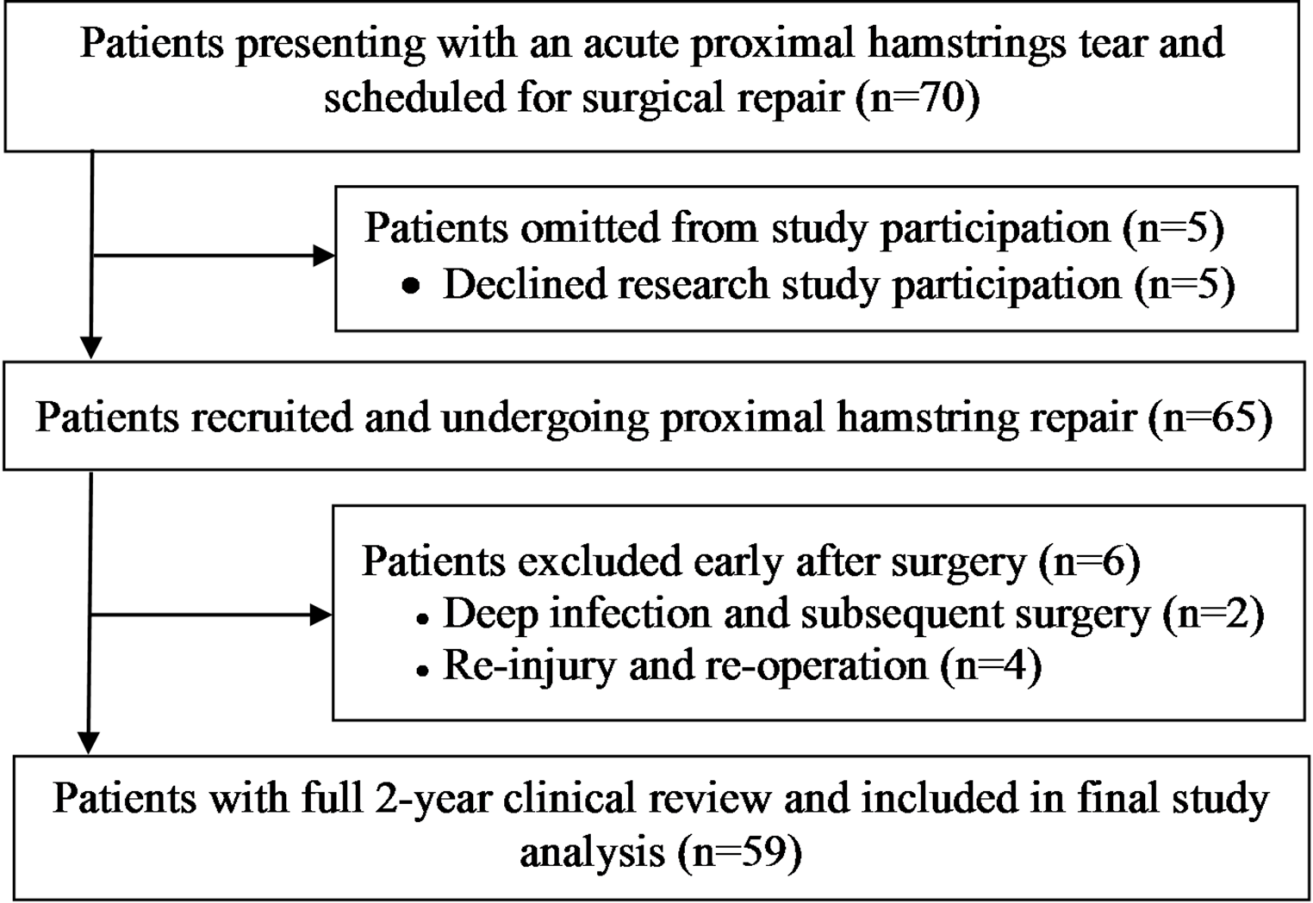
Flowchart demonstrating patients presenting with an acute proximal hamstrings tear, repair, those recruited and subsequently omitted from the final study analysis

This study prospectively screened 70 patients with acute proximal hamstring tears and subsequently scheduled for surgical repair by one of three orthopaedic surgeons, between November 2020 and March 2023. Of these, 59 were included in the current analysis (Fig. 1). All patients underwent proximal hamstring repair for ‘acute’ tears. While several definitions have been described including a < 4 weeks [15, 17, 25], < 6 weeks [2, 6], < 8 weeks [23] and < 3 months [24], the current study defined acute repair as those within 6 weeks of the designated injury. Otherwise, further criteria for surgery included skeletally maturity, symptoms associated with the proximal hamstring rupture, and radiological diagnosis confirmed in all patients via Magnetic Resonance Imaging (MRI). This prospective study was approved by the institutional Human Research Ethics Committee (HREC).

### Surgical procedures and rehabilitation

All three surgeons that recruited patients adopted a similar surgical technique, with surgery undertaken with the patient in prone and under a general anaesthetic. Pre-operative antibiotics were provided, while aseptic protocols were undertaken including hair removal, pre-wash and placement of a betadine-soaked gauze into the perineal area. A transverse skin incision was used within the gluteal fold, with sub-gluteal dissection down to the ischium performed with careful visualization, formal identification and neurolysis. Tendon mobilization was undertaken to ensure adequate reduction under minimal tension. The lateral wall of the ischium was prepared, and surgical repair was undertaken using ≥ 3 anchors in a double-row configuration. The surgical field was washed, with the wounds closed in layers and the skin closed with absorbable subcuticular sutures.

Post-operatively, patients were instructed to partially weight bear with crutches for the first two weeks to allow pain relief, comfort and wound healing. At two weeks post-surgery, patients were generally permitted to wean from crutches as tolerated, with unaided ambulation routinely achieved 2–4 weeks post-operatively. While therapist-led rehabilitation was generally initiated from this time, initially focusing on hip and knee range of motion (ROM) exercises, isometric quadriceps and gluteal exercises, and gait retraining, the progression of strength and conditioning activities, along with the introduction of various recreational and/or sporting activities, was undertaken under the direction of the patient’s own out-patient therapist.

### Clinical outcome measures

Firstly, the Perth Hamstring Assessment Tool (PHAT) [4] and Lower Extremity Functional Scale (LEFS) were assessed pre-surgery and at 2-years post-surgery. An increased commitment to the use of patient-reported outcome measures (PROMs) such as the PHAT and LEFS, as well as isokinetic strength testing, have been recommend in the assessment of patients undergoing proximal hamstring repair [22]. Specifically at 2-years post-surgery, a patient satisfaction questionnaire was also employed to assess the level of satisfaction with the surgery overall. A Likert response scale was employed with descriptors ‘Very Satisfied’, ‘Somewhat Satisfied’, ‘Somewhat Dissatisfied’ and ‘Very Dissatisfied’.

### Predictor variables

A range of variables were collected and available for inclusion in the predictive models. Firstly, patient demographics selected included age, sex and body mass index (BMI). Pre-operative injury/surgery characteristics included the time from injury to surgery (days), as well as the specific mechanism of injury. For the current study, mechanism of injury was graded as ‘sporting activities’ (such as Australian Rules Football, soccer, rugby and tennis), ‘recreational activities’ (such as running, skateboarding and roller-skating) and those experienced during ‘work activities, activities of daily living (ADLs) and motor vehicle accidents. The pre- and post-operative Tegner Activity Score (TAS) was assessed and included, as was the post-operative peak isokinetic hamstring strength torque (normalised to body weight). Peak isokinetic knee flexor torque was assessed at an angular velocity of 90°/s using an isokinetic dynamometer (Isosport International, Gepps Cross, South Australia).

Pre-operative MRI-based factors included the degree of semimembranosus (SM) and/or conjoint tendon (CT, semitendinosus & biceps femoris) retraction (mm), as well as the Proximal Hamstring Objective Magnetic Resonance Imaging Score (PHOMRIS) for the SM and CT hamstring components as previously reported [8]. The degree of retraction was initially reported in all pre-operative MRIs, though both retraction and the PHOMRIS were subsequently scored by an independent and experienced musculoskeletal radiologist. Furthermore, inter-observer reliability was assessed for the PHOMRIS using the Spearman’s rank order (rho) correlation for each proximal hamstring component (SM rho = 0.810, p *<* 0.001; CT rho = 0.842, p *<* 0.001), by a second independent radiologist scoring 30 of the pre-operative MRI studies. Intra-observer reliability was assessed by the primary radiology assessor re-scoring 30 MRI studies (SM rho = 0.815, p *<* 0.001; CT rho = 0.902, p *<* 0.001).

### Statistical analysis

Descriptive statistics were performed for baseline clinical scores, patient and surgical characteristics, and 2-year clinical scores. Paired t-tests were used to estimate the change in clinical outcomes from baseline to 2 years after surgery. The minimal important change (MIC) for the PHAT was determined by using the 0.5 SD of the baseline values of the PHAT score [21]. The percentage of patients reaching the MIC level at 2 years post-surgery was reported. Univariable and multivariable linear regressions were undertaken for the 2-year PHAT score, while binary logistic regression analyses were employed for 2-year overall satisfaction with the surgery (‘Very Satisfied’ versus all other satisfaction responses). For each regression model, potential predictors were first evaluated univariably, and those displaying associations with outcomes at *p* ≤ 0.20 were included in a multivariable regression model. In the final step, non-significant variables (*p* > 0.05) were removed from the initial multivariable model one at a time. In the linear regression model for the 2-year PHAT score, Beta coefficients, along with their 95% confidence intervals, were calculated to quantify the influence of each predictor on the dependent variable, with standardized Beta values (β) included in the final multivariable model. For the binary logistic regression analysis, each predictor variable was expressed as odds ratios (OR) and 95% confidence intervals. All analyses were carried out using JASP software (Version 0.17, JASP Team, University of Amsterdam).

## Results

Of the 70 patients prospectively screened for study participation, 59 were included in the current analysis (Fig. 1). Reasons for study exclusion included not wanting to participate in the research (*n* = 5), early post-operative deep infection that required further surgery (*n* = 2), or early (*n* = 2, both within the first three post-operative months) or mid/late (*n* = 2, both within 9–12 months post-surgery) stage re-injury and re-operation. Of those remaining, all had full clinical follow-up to 2-years post-surgery (Fig. 1).

Of the included cohort, baseline clinical scores, participant characteristics, and surgical parameters are presented in Table 1. Mean improvement in PHAT scores from baseline to 2 years post-surgery was 47.1 points (95% CI, 41.9 to 52.3; *p* < 0.001). The MIC for the PHAT score was 8.6 points at 2 years post-surgery, and 68 (97.1%) patients improved by at least the MIC from baseline to 2 years. The LEFS improved by 44.5 points (95% CI, 39.9 to 49.1; *p* < 0.001), while the TAS improved by 2.6 points (95% CI, 2.1 to 3.2; *p* < 0.001). Normalized peak knee flexor torque improved by 0.16 Nm/Kg from 6 months to 2 years post-surgery (95% CI, 0.11 to 0.21; *p* < 0.001). Of the 59 patients included, 46 (78.0%) were ‘very satisfied’ with their 2-year overall outcome, 11 (18.6%) were ‘somewhat satisfied’ and 2 (3.4%) were ‘somewhat dissatisfied’.

**Table 1:** Patient demographics, along with injury and surgery characteristics, and pre-operative magnetic resonance imaging (MRI) variables, of the patient cohort undergoing proximal hamstring repair and included in the final analysis. Shown are means (SD) or n (%)

	Baseline	2 years
*Participant characteristics*		
Age, y	48.6 ± 13.3	
Sex – Female	25 (42.4%)	
BMI, kg/m^2^	27.6 ± 4.7	
Time from injury to surgery, days	25.6 ± 12.0	
*Injury mechanism*		
Sporting Activities	23 (39.0%)	
Recreational Activities	11 (18.6%)	
Work/ADLs/MVA	25 (42.4%)	
*MRI tissue characteristics*		
Semimembranosus retraction, mm	43.6 ± 29.4	
Conjoint tendon retraction, mm	44.2 ± 25.2	
Semimembranosus score (1–7)	6.2 ± 1.7	
Conjoint tendon score (1–7)	6.5 ± 1.5	
*Clinical outcome*		
Perth Hamstring Assessment Tool (PHAT)	35.9 ± 17.1	83.3 ± 12.6
Lower Extremity Functional Scale (LEFS)	30.1 ± 17.1	74.6 ± 6.5
Tegner Activity Score (TAS)	2.7 ± 2.1	5.3 ± 1.7
Peak Knee Flexor Torque, Nm/Kg^− 1^		1.30 ± 0.30
Satisfaction – ‘very satisfied’ (vs. all other responses), n (%)		46 (78.0%)

ADLs = activities of daily living; MVA = motor vehicle accident; MRI = magnetic resonance imaging

### PHAT Score

Univariable and multivariable linear regression models are shown in Table 2. In the univariable analysis, age, baseline TAS, a sport and recreation injury mechanism, and 6-month normalized peak knee flexor torque were all significantly associated with the 2-year PHAT. While greater knee flexor strength was associated with better outcomes univariably (B = 1.16 per 0.1 Nm/kg; *p* = 0.030), this effect was not maintained in the multivariable analysis (*p* = 0.918), when adjusting for injury mechanism. The final multivariable model consisted of baseline TAS and a sport and recreation injury mechanism (Table 2). Proximal hamstring repair as a result of an injury sustained during sport/recreation was associated with a 9.38 point higher PHAT score at 2 years compared to ADL/work mechanisms (95% CI, 3.58 to 15.18; *p* = 0.002). Additionally, a higher baseline TAS was also independently associated with a better 2-year PHAT (B = 1.77; *p* = 0.015). The adjusted R² for this model was 0.179.

**Table 2:** Univariable and multivariable linear regression models for the 2-year Perth Hamstring Assessment Tool (PHAT) Score

Predictor Variable	Univariable		Multivariable (Adjusted *R*^2^ = 0.259)		Final Multivariable Model (Adjusted *R*^2^ = 0.243)		
	B (95% CI)	*p* value	B (95% CI)	*p* value	B (95% CI)	Standardized β	*p* value
Baseline PHAT	0.10 (-0.10 to 0.29)	0.326					
Baseline TAS	2.16 (0.63 to 3.68)	0.006	1.58 (0.14 to 3.06)	0.047	1.70 (0.26 to 3.13)	0.287	0.021
Age, y	-0.35 (-0.59 to 0.12)	0.004	-0.11 (-0.36 to 0.14)	0.442			
Sex – Female	-1.13 (-7.83 to 5.59)	0.738					
BMI, kg/m^2^	0.02 (-0.70 to 0.74)	0.964					
Time from injury to surgery, days	-0.12 (-0.40 to 0.16)	0.385					
Injury mechanism							
Sport/Recreation vs. ADLs/Work	11.19 (5.16 to 17.21)	**< **0.001	8.16 (1.62 to 14.70)	0.015	9.85 (3.94 to 15.76)	NA	0.001
Semimembranosus retraction	0.05 (-0.06 to 0.17)	0.935					
Semimembranosus score	-0.80 (-2.74 to 1.15)	0.414					
Conjoint tendon retraction	0.01 (-0.13 to 0.14)	0.929					
Conjoint tendon score	-1.97 (-4.17 to 0.23)	0.079	-1.70 (-3.72 to 0.32)	0.097			
6-month Knee Flexor Torque (Nm/kg)	1.16 (0.12 to 2.20)*	0.030	0.06 (-1.60 to 1.18)	0.918			

PHAT = Perth Hamstring Assessment Tool, TAS = Tegner Activity Scale, BMI = body mass index, ADLs = activities of daily living. *Per 0.1 Nm/kg

### Overall satisfaction

The binary logistic regression analysis for overall “complete” satisfaction at 2 years is shown in Table 3. Time from injury to surgery (*p* = 0.160) and SM retraction (*p* = 0.073) were factors univariably associated (*p* < 0.20) with overall satisfaction. In the final model, no individual variable reached statistical significance (*p* < 0.05). These predictors accounted for 14.1% of the variance in being ‘very satisfied’ with the surgical outcome at 2 years (Nagelkerke R² = 0.141).

**Table 3:** Univariable and multivariable binary logistic regression models for 2-year overall “complete” satisfaction with proximal hamstring repair surgery

Predictor Variable	Univariate			Multivariable (Nagelkerke *R*^2^ = 0.141)		
	B (SE)	OR (95% CI)	*p* value	B (SE)	OR (95% CI)	*p* value
Baseline PHAT	-0.01 (0.02)	0.99 (0.95 to 1.03)	0.564			
Baseline TAS	0.05 (0.16)	1.05 (0.77 to 1.44)	0.754			
Age, y	-0.01 (0.02)	0.99 (0.94 to 1.04)	0.707			
Sex – Female	0.21 (0.64)	1.23 (0.35 to 4.34)	0.747			
BMI, kg/m^2^	-0.02 (0.07)	0.98 (0.86 to 1.12)	0.789			
Time from injury to surgery, days	-0.04 (0.03)	0.96 (0.91 to 1.02)	0.160	-0.04 (0.03)	0.96 (0.91 to 1.02)	0.208
Injury mechanism						
Sport/Recreation vs. ADLs/Work	0.60 (0.63)	1.82 (0.53 to 6.28)	0.347			
Semimembranosus retraction	0.26 (0.16)	1.03 (1.00 to 1.06)	0.073	0.02 (0.01)	1.02 (1.00 to 1.05)	0.092
Conjoint tendon retraction	0.01 (0.01)	1.00 (0.98 to 1.03)	0.763			
6-month Knee Flexor Torque (Nm/kg)	-0.01 (0.10)*	0.99 (0.81 to 1.21)	0.913			

PHAT = Perth Hamstring Assessment Tool, TAS = Tegner Activity Scale, BMI = body mass index, ADLs = activities of daily living. *Per 0.1 Nm/kg

## Discussion

The most important findings of the current study were that clinical scores improved from baseline to 2 years in patients undergoing proximal hamstring repair for acute tendon ruptures. Furthermore, the pre-operative TAS and a sport and recreation injury mechanism were associated with the 2-year PHAT, though no individual variables were significantly associated with being ‘very satisfied’ with the 2-year surgical outcome.

The clinical PROMs employed in the current study significantly improved from baseline to 2 years post-surgery, as did the normalized peak isokinetic knee flexor torque from 6 months to 2 years, in support of the first hypothesis. This included a mean improvement of 47.1, 44.5 and 2.6 points for the PHAT, LEFS and TAS, respectively. While a recent systematic review recommended an increased commitment to the use of the PHAT and LEFS in those undergoing proximal hamstring repair, as well as isokinetic strength testing [22], to the best of the authors’ knowledge minimal clinically important differences (MCIDs) have not been reported for these PROMs in this cohort. In the current study, 97% of patients improved by at least 8.6 points in the PHAT by 2 years, calculated as the MIC, indicating a clinically relevant improvement in the majority of patients. Nonetheless, the improvement in clinical scores and strength in patients undergoing repair for proximal hamstring avulsions has been previously reported [5, 10, 13, 14]. The improvement in clinical scores in the current study was reflected in the satisfaction scores, with 96.6% of patients satisfied (and 78% being ‘very satisfied’). High satisfaction rates have been reported after proximal hamstring repair in other studies and reviews, ranging from 88 − 10% [5, 14, 23].

Factors that were univariably associated with the 2-year PHAT included age, a sport and recreation injury mechanism, pre-operative TAS and 6-month normalized peak knee flexor torque, though the final multivariable model consisted of baseline TAS and a sport and recreation injury mechanism. While this supported the second hypothesis, the modest adjusted R² indicates relatively low explanatory power, with a large proportion of outcome variability clearly unexplained. Furthermore, no variable was significantly associated with being ‘very satisfied’ with 2-year surgical outcome. However, as reported a total of 46 patients (78%) were ‘Very Satisfied’ and 11 patients (19%) were ‘Somewhat Satisfied’, with 57 of 59 patients (97%) therefore satisfied with their outcome. Therefore, it is likely that the high number of satisfied patients likely suggests a ceiling effect and low statistical power to detect specific predictors of being satisfied.

Bowman et al. [6] could not identify any relationships between patient demographics, medical comorbidities, tear characteristics and repair technique, with clinical outcomes at a minimum 1-year after proximal hamstring repair. Lefèvre et al. [20] reported significant functional improvement in patients that underwent proximal hamstring repair at a mean follow up period > 4 years, with younger (< 50 years of age) versus older patients demonstrating a greater magnitude of improvement in the Parisian Hamstring Avulsion Score (PHAS), the TAS and the University of California, Los Angeles (UCLA) scale. Best et al. [3] reported that a surgical delay and female sex were associated with a worse functional outcome (PHAT) at a minimum 6 months after proximal hamstring repair. While return to sport (RTS) status was not assessed in the current study, a recent review reported that elite athletes are more likely to return when compared with non-elite [7]. In a study on professional athletes, being male, those undergoing surgery for isolated semimembranosus tears, or surgery for complete tendon ruptures without evidence of an empty footprint, were all predictive of a better post-operative level of sporting competition [18]. While numbers in the current study did not permit an analysis on re-injury, a recent study reported that patient age and a non-sporting injury mechanism were associated with a higher risk of re-injury in patients undergoing repair of acute proximal hamstring tears [9].

Of interest, the current study was unable to identify an association between pre-operative MRI-based factors, including the degree of tendon retraction or the PHOMRIS for the SM and CT hamstring components [8], on 2-year clinical outcome. Best et al. [3] reported that the degree of pre-operative stump retraction on MRI was not associated with outcome at a minimum 6 months after proximal hamstring repair. However, Fenn et al. [11] reported an improvement in PROMs after proximal hamstring repair, though further investigated differences based on open and endoscopic repair, and tear type (grade 1, incomplete tear with the involvement of 1 or 2 tendons; grade 2, complete tear with the involvement of 3 tendons and ≤2 cm retraction, and; grade 3, complete tear with the involvement of 3 tendons and >2 cm retraction). Those with grade 3 tears demonstrated significantly lower rates of 2-year Patient Acceptable Symptom State (PASS) achievement in PROMs, with endoscopic (versus open) repair for grade 2 tears demonstrating better outcomes. It should be noted that the current study investigated factors (including pre-operative MRI variables such as the degree of tendon retraction) that were associated with the outcome of surgery for acute (≤ 6 weeks) tears, and these pre-operative MRI variables may play a larger role in the context of repair for more chronic tears.

Several limitations are acknowledged in the current study. Firstly, the size of the cohort (*n* = 59) included was limited by those presenting within our research institution over the period, as well as those with the full suite of clinical outcomes and predictive variables collected for inclusion in the models. Furthermore, as outlined the limited explanatory power of the multivariable model suggests that a large proportion of outcome variability in the PHAT remains unexplained, while the high percentage of patients reporting satisfaction with their outcome likely suggests a ceiling effect and low statistical power to detect specific predictors of being ‘Very Satisfied’. It is also acknowledged that the study included those undergoing surgery for acute repairs and via an open surgical approach, and the outcomes cannot be extrapolated to those undergoing endoscopic repair or surgery for chronic injuries. The current study employed the PHAT and LEFS which have been recommended for use in those undergoing proximal hamstring repair [22], though other PROMs have been previously employed including the PHAS, the Proximal Hamstring Injury Questionnaire (PHIQ), the Sydney Hamstring Origin Rupture Evaluation (SHORE) and the International Hip Outcome Tool (iHOT-12) [12, 19].

As the clinical relevance, the current study represents the most comprehensive analysis seeking to investigate a robust array of factors and their association with post-operative clinical outcome, including patient, injury/surgical (including MRI-based variables) and post-operative (such as flexor strength) variables. The study demonstrated improved outcomes at 2-years following proximal hamstring tendon repair for acute tendon avulsions. While the pre-operative TAS and a sport and recreation injury mechanism were associated with the 2-year PHAT, no individual variables were significantly associated with being ‘very satisfied’ with the 2-year surgical outcome. This information may permit more tailored education and discussion with patients, along with the setting of realistic mid-term expectations on outcome.

## Conclusion

Clinical scores improved significantly from baseline to 2 years after proximal hamstring repair. While pre-operative TAS and a sport/recreation injury mechanism were associated with the 2-year PHAT, no variables were associated with being ‘very satisfied’ with the 2-year outcome. This information may permit more tailored education and discussion with patients around their individual presentation and the association these variables may have with post-operative outcome, along with the setting of realistic mid-term expectations on outcome.

## Data Availability

No datasets were generated or analysed during the current study.

## Abbreviations

MRI: Magnetic resonance imaging
BMI: Body mass index
SM: Semimembranosus
CT: Conjoint tendon
